# Reassessing the cryopreserved human amniotic membrane's low immunogenicity due to advances in histocompatibility

**DOI:** 10.1016/j.mtbio.2025.102619

**Published:** 2025-12-01

**Authors:** Jean-Baptiste Baudey, Lauriana Solecki, Christophe Picard, Pascal Pedini, Bastien Mathéaud, Alain Coaquette, Isabelle Jollet, Pauline Jamain, Lucas Hubert, Adeline Desanlis, Xavier Lafarge, Florelle Gindraux

**Affiliations:** a Établissement Français du Sang Provence Alpes Côte d’Azur-Corse, Laboratoire d'immunogénétique, F-13005, Marseille, France; b Aix-Marseille Université, CNRS, EFS, ADES UMR 7268, F-13005, Marseille, France; c Centre Hospitalier Universitaire de Besançon, Service d’Ophtalmologie, F-25000, Besançon, France; d Université Marie et Louis Pasteur, UR 4662 Soins Intégrés, Nanomédecine, IA et Ingénierie pour la Santé (SINERGIES), F-25000, Besançon, France; e Nouvel Hôpital Civil CHRU Strasbourg, Service d’Ophtalmologie, F-67000, Strasbourg, France; f Centre Hospitalier Universitaire de Besançon, Service de virologie, F-25000, Besançon, France; g Établissement Français du Sang Nouvelle-Aquitaine, Laboratoire d’Histocompatibilité, F-86012, Poitiers, France; h Établissement Français du Sang Bourgogne Franche-Comté, Activité d'ingénierie cellulaire et tissulaire, F-25000, Besançon, France; i Établissement Français du Sang Nouvelle-Aquitaine, Laboratoire d'ingénierie tissulaire et cellulaire, F-33075, Bordeaux, France; j Inserm U1211 Maladies Rares: Génétique et Métabolisme, Université de Bordeaux, F-33075, Bordeaux, France; k Centre Hospitalier Universitaire de Besançon, Service de Chirurgie Maxillo-Faciale, Stomatologie et Odontologie Hospitalière, F-25000, Besançon, France

**Keywords:** Human amniotic membrane, Biological scaffold, Immunogenicity, HLA, Luminex, MAIPA

## Abstract

Human amniotic membrane (hAM) is a biocompatible scaffold with suitable mechanical properties (permeability, stability, elasticity, flexibility, resorbability and transparency), that is rich in stem cells and growth factors and is used for tissue repair and regenerative processes. It is routinely used in ocular surgery as it promotes epithelialization, supports cell adhesion and proliferation, and enhances wound healing. It exhibits both immunomodulatory and immunoregulatory properties, contributing to its anti-inflammatory and low immunogenicity risk profile.

hAM transplantation is considered unlikely to trigger an immune response against human leukocyte antigens (HLA) and is generally regarded as minimally or non-immunogenic. This conclusion is primarily based on clinical observations, and a single study published 40 years ago that investigated subcutaneous hAM grafting—a procedure that does not reflect current clinical applications.

Since then, there has been no laboratory evidence that hAM transplantation is immunologically safe. Notably, the production of anti-HLA antibodies following hAM transplantation has not been investigated, despite the tremendous improvements in the sensitivity of detection techniques. The recent application of hAM to more vascularized areas could raise concerns about potential immune responses.

In this perspective paper, we summarize the limited and sometimes contradictory data regarding hAM immunogenicity. Then, we describe the methods we use to assess whether immune responses are triggered after hAM grafting in current clinical indications.

## Graphical abstract

**Figure:**
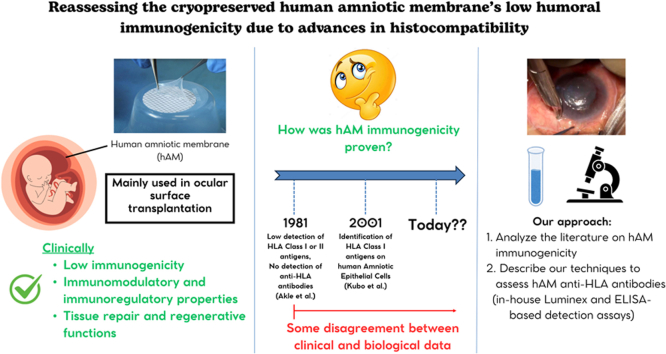

## 1 Introduction

**Fig. 1:**
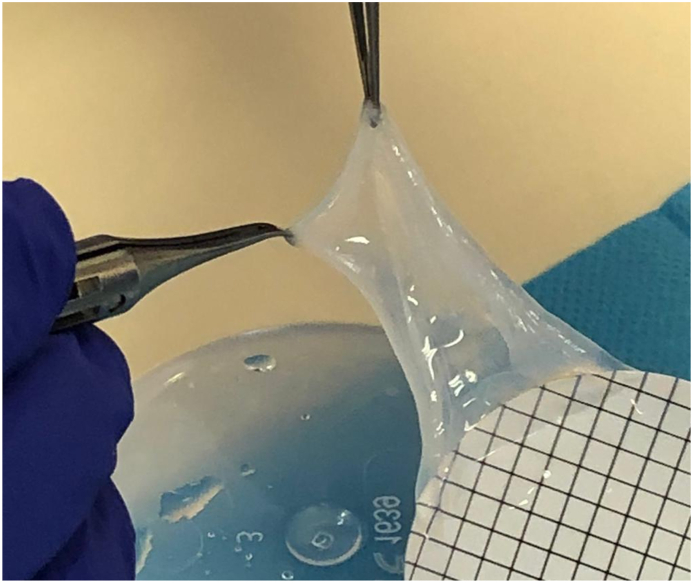
Human amniotic membrane (hAM) used for clinical application.

The human amniotic membrane (hAM) corresponds to the wall of an embryo/fetal annex called the amnion or amniotic sac, which encloses the amniotic cavity and contains amniotic fluid (Fig. 1) [1]. Histologically, it is composed of an epithelial monolayer containing human amniotic membrane epithelial cells (hAECs), a thick basement membrane and an avascular stroma containing human amniotic membrane mesenchymal stromal cells (hAMSCs). When used as a graft, it provides mechanical support and has the ability to reduce scarring and inflammation during healing [2,3]. Its cellular components, cytokines and growth factors also have anti-inflammatory, anti-microbial, and either anti-angiogenic or angiogenic properties (depending on the application), as well as analgesic properties [[2], [3], [4], [5], [6]].

Fresh hAM has been widely used since 1910, initially for skin repair [7]. Its application has since expanded significantly to ophthalmology [8] and then to various conditions requiring soft tissue healing [9].

This allograft is considered as having low immunogenicity [[10], [11], [12], [13]] with a high margin of clinical safety given the small number of serious adverse reactions observed despite the high number of transplants [13]. No biological testing to assess hAM immunogenicity is currently required [6,[14], [15], [16], [17], [18]]. In France (as in other European countries), its routine approved use – mainly as a scaffold – is limited to ophthalmologic indications, with the eye being considered as an immune privileged site. Other indications are subject to ethical and regulatory authorizations, where monitoring for a potential inflammatory reaction is recommended but not required. In Germany, hAM is approved for use in orbital, oral and maxillofacial surgery, gynecological surgery, and as a temporary skin substitute. In the USA, FDA-approved uses of hAM include wounds, ulcers, burns, adhesions, and skin injuries. It is also widely used in gynecology, plastic surgery, gastrointestinal surgery, traumatology, neurosurgery, and ophthalmology [19].

The conventional storage methods for hAM are cryopreservation, lyophilization (or freeze-drying), and air-drying (dehydration) [19]. Cryopreservation requires the use of a cryoprotectant to ensure cell viability survival. Lyophilization involves removing water from hAM through sublimation. During the air-drying or dehydration process, the hAM is maintained at ambient temperature within a laminar flow hood, where it is exposed to the atmosphere for varying durations. Lyophilization and air-drying are typically followed by gamma irradiation to ensure the hAM is safe. This combination is often referred to as "hyperdried hAM". The majority of these processes were developed with the specific intention of facilitating hAM storage and have been patented by academic laboratories and commercial entities [20]. Decellularization and devitalization processes have been developed, with claims that they ensure hAM safety and prevent immune rejection, while its safety was already broadly recognized [19].

hAM fulfils the main requirements for tissue engineering—including serving as a three-dimensional, biodegradable, and biocompatible scaffold that contains growth factors and stem cells [21,22]. It has been widely used as a scaffold for tissue engineering purposes in clinical settings but has also been used in advanced forms, such as hAM-based hydrogels, bioinks, nanoparticles, and hybrid scaffolds [22,23]. Recently, new multilayer composite-based hAMs have demonstrated significant benefits for additive manufacturing technologies, particularly electrospinning, in expanding their applications in tissue engineering and clinical use [24].

Sporadic, contradictory, and poorly documented results have been reported since 1940, when de Rötth et al. reported a low clinical success rate after using fresh hAM and chorion together for the plastic repair of conjunctival defects [8]. Since the 1980s, several studies have been conducted on human leukocyte antigen (HLA) expression by hAM, providing new information about its immunogenicity.

In this perspective paper, we did a comprehensive and critical review of the existing literature on hAM immunogenicity. Analyzing past findings gave us an up-to-date and nuanced perspective on the limited and occasionally conflicting data and should help readers understand why some historical results are conflicting. Importantly, while some studies reported contradictory results, none has ever challenged the established non-immunogenic profile of hAM. In addition, drawing on our extensive experience in HLA detection, [[25], [26], [27], [28], [29], [30], [31], [32], [33], [34], [35]], we describe two complementary methodological approaches designed to assess hAM humoral immune response, encompassing both classical and non-classical HLA systems. The first method – the Luminex Single Antigen (Ag) assay – is a well-established and highly sensitive technique for precise detection and identification of antibodies (Abs) directed against classical HLA molecules. The second is a novel and innovative protocol specifically developed to evaluate the humoral immune response of non-classical HLA-G Ags. These approaches offer novel experimental insights and a methodological framework for future studies. This will allow researchers to document hAM tolerance when it is grafted into deep and vascularized tissues or in patients with prior immunization that could negatively affect graft efficacy.

## 2 Immunogenicity

a) General concepts in HLA class II and class II

The major histocompatibility complexes (MHC), or the HLA system, play a major role in histocompatibility and graft immunogenicity because of their polymorphisms and their role in the immune response. The HLA system includes HLA class I molecules that associate with β2 microglobulin (β2m) and HLA class II molecules (DR, DP and DQ). Among the HLA class I Ags, the classical molecules of the HLA-A, -B, and -C loci (HLA class Ia) are more polymorphic than the non-classical molecules HLA-E, -F, -G (HLA class Ib). Immunization against classical HLA Ags is frequent in organ transplantation and is associated with a risk of humoral rejection. It is therefore closely monitored using Luminex technique. Despite their lower polymorphism, non-classical Ags have several identified variants and could theoretically also induce or be the target of allo-Abs, like other more polymorphic Ags, in the context of a mismatched graft.

b) Lack of hAM immunogenicity

The lack of immunogenicity is one of the most prominent and well-documented characteristics of hAM and its derived cells, contributing to a state of immunological tolerance [36]. This tolerance arises from passive mechanisms: absence or low expression of immunogenic surface markers such as CD80, CD86 or HLA-DR [37]; and active mechanisms involving the secretion of soluble factors, hAECs derived exosomes, direct immune modulation and expression of mesenchymal stem cells markers such as CD44 or CD73 [38].

hAM and its derivatives, including hAECs and hAMSCs, promote immune tolerance through several pathways: they can induce regulatory T-cells (Tregs), inhibit effector T-cell proliferation, and reduce inflammatory cytokine secretion [36,39]. In addition, hAM-derived cells exhibit potent immunomodulatory properties, meaning they can regulate or balance immune functions through direct cell contact or the release of soluble mediators [40]. For example, hAECs and their conditioned medium can inhibit T-cell proliferation even in the absence of direct contact, underscoring the key role of soluble immunosuppressive factors [41]. Indoleamin 2,3-dioxygenase (IDO) secretion regulates T-cell proliferation and Th17 differentiation [36]. Both hAECs and hAMSCs suppress T-cell activation and proliferation in response to allo-Ags and in various immune stimulation models, in a dose-dependent manner. This has been demonstrated after T-cell exposure to allo-Ags in the presence of hAM-derived stem cells, either following CD3/CD28 stimulation or in classical mixed lymphocyte reaction assays [42,43].

Other immune checkpoint molecules, such as programmed death ligands 1 and 2 (PD-L1 and PD-L2), are also expressed by hAMSCs and by syncytiotrophoblasts in the placenta. These molecules are similarly implicated in the induction of immune tolerance by hAM-derived cells [44], and can be produced by hAECs even without IFN-γ stimulation [45].

Furthermore, hAM secretes several anti-inflammatory mediators, including interleukin-10 (IL-10), prostaglandin E2 (PGE2), and macrophage migration inhibitory factor (MIF), which modulate both innate and adaptive immune responses [37]. These factors promote the polarization of macrophages toward an M2 phenotype, the induction of Tregs, and the inhibition of dendritic cell maturation, thereby limiting Ag presentation and preventing excessive immune activation. Endocrine secretion of cytokines has an inhibitory effect on a wide variety of immune effector cells. Some of these molecules are produced in response to stimulation by pro-inflammatory signals, reinforcing the concept of an active immunosuppressive mechanism [46]. Rather than inducing broad immunosuppression, hAM establishes a controlled immunoregulatory microenvironment that favors tissue repair and graft tolerance, consistent with the physiological role of the amnion in maintaining fetal-maternal immune homeostasis [[47], [48], [49], [50]].

Regarding humoral immunity, hAM-derived cells express very low levels of classical immune markers. They typically lack major MHC class II molecules (such as HLA-DR) and hematopoietic markers (CD80, CD86), thereby minimizing their ability to activate B cells or elicit Ab production [37,51].

Therefore, hAM and its derived cells exert potent immunoregulatory effects that contribute to maintaining local immune tolerance after allogeneic transplantation.

c) Specific features of HLA-G

A crucial contributor to this immunomodulatory ability is the expression of HLA-G, a non-classical, tolerogenic HLA molecule [52]. HLA-G expressed by hAECs has the ability to impair T cell proliferation through direct contact or through HLA-G expressing extravesicules [53].

HLA-G is well known for its immunomodulatory properties. It enables fetal tolerance by the mother despite their partial HLA incompatibility and the invasion of the uterine wall. It is highly expressed during pregnancy in the trophoblast, where its interaction with Killer-cell immunoglobulin-like receptors (KIR) on maternal natural killer (NK) cells facilitates immune tolerance [[54], [55], [56]]. Additionally, HLA-G has the capacity to bind Ig-like transcript 2 (ILT2) (also known as leukocyte Ig-like receptor 1, CD85j, and LILRB1) and Ig-like transcript 4 (ILT4) (also known as leukocyte Ig-like receptor 2, CD85d, and LILRB2) receptors, which are expressed by many leukocyte subsets. This broad immunomodulatory effect underlies its now well-documented role in promoting tolerance after organ transplantation. In this context, HLA-G expression by graft cells and its soluble form in the plasma of transplant recipients has been associated with a reduced risk of rejection [[57], [58], [59]].

It has long been known that HLA-G is expressed by hAM cells, as demonstrated by studies at both the mRNA and protein levels [56], including its soluble form in the amniotic fluid and cell culture supernatants [60]. Moreover, its expression can be induced by inflammatory cytokines [46].

HLA-G expressed on hAM has been shown to exert an immunosuppressive effect on specific cell types, such as NK cells and monocytes [61], and more recently, on T lymphocyte proliferation. This immunosuppressive process is reversible and can be neutralized by the use of anti-HLA-G Abs [53].

Thus, HLA-G appears to play a crucial role in preventing rejection of hAM grafts [52], conferring immune-privileged status to this tissue and forming the basis for its therapeutic use [62].

Classical HLA molecules are highly immunogenic, which is a major challenge in organ transplantation. In contrast, HLA-G is far less polymorphic, with only about 100 alleles described. This is said to reduce the frequency of incompatibility and therefore of immunological conflict. However, anti-HLA-G Abs have recently been identified in autoimmune conditions and also in healthy individuals [63]. Consequently, one might question whether hAM transplantation could induce anti-HLA-G immunization, given the strong physiological expression of HLA-G in this tissue.

So far, anti-HLA-G immunization has not been explored following hAM transplantation. However, to fully evaluate the immunogenicity of hAM transplantation, and given the spectrum of HLA molecules expressed by hAM, we believe it is crucial to supplement the detection of classical anti-HLA Abs with an investigation into anti-HLA-G Abs. These Abs could potentially have detrimental effects by blocking the binding of HLA-G to its receptors, thereby impairing its immunomodulatory capacities.

## 3 Conflicting historical data on HLA class I and class II detection in hAM

In the 1980s, Akle and colleagues provided evidence that hAM was a safe allograft. They detected no anti-HLA Abs in the serum of four volunteers who had received a subcutaneous graft of fresh hAM [10]. In this context, they investigated the expression of HLA Ags in hAM and did not detect HLA class I (A, B, C) or II Ags using immunofluorescence on freshly collected or cultured hAEC. The scientific community has long considered this to be a landmark study supporting its non-immunogenicity, as reflected by the fact that their article has been cited 728 times. However, weak HLA Ag expression was identified when more sensitive radiobiological techniques were applied [11].

During the following decade, Akle's results were rapidly challenged by the scientific community who focused on HLA detection from whole amnion or isolated cells [56,60,64,65]. Using high-affinity monoclonal Abs and the sensitive immunoperoxidase method, Hsi et al. identified HLA class I Ags on the amniotic epithelium of cryopreserved hAM [65]. They specified that the level of expression was different between cells from various parts of the amnion, with the strongest reactivity consistently found in the amniotic epithelium at the edge of the placenta. Using an avidin-biotin immunoperoxidase staining system and analyzing HLA class I mRNA through Northern blotting and in situ hybridization, Hunt et al. suggested that amnion cells transcribed HLA class I genes and were capable of synthesizing class I H chains, although expression appeared modulated by extrinsic regulatory molecules [64]. Additionally, the expression of HLA class I Ags by trophoblast cells varied based on the stage of trophoblast cell differentiation, the anatomic location of the cell, and whether the trophoblast cell was exposed to decidual cells or maternal blood. Using immunostaining and fluorescence-activated cell sorting (FACS) analysis, Houlihan et al. found strong expression of HLA class Ib Ag, but limited and variable expression of polymorphic HLA class Ia only on amnion cells isolated from fresh hAM [56]. Additionally, Northern blotting using locus-specific probes demonstrated that amnion expresses two class Ib genes: HLA-E and HLA-G. They concluded that amnion at term appeared to express class Ib antigens and, to a lesser extent, class Ia Ags. Therefore, HLA-G was expressed in two extrafetal epithelia: amnion and trophoblast. Using immunohistochemical, biochemical and molecular biology methods, and a set of specific Abs against different HLA class I determinants, Hammer et al. highlighted the frequent detection of HLA-A, -B, -C, and -G proteins on hAECs of term placenta. [60]. They found considerable variations in the levels and types of HLA class I molecules expressed from one placenta to another. Moreover, the expression level depended on where the sample was collected within the amnion and could differ from cell to cell. Using flow cytometry, positive expression for HLA-A,B,C, but no expression for HLA-DR from both passage 2–4 hAMSCs and hAECs, was observed [66,67]. Later, passage 1 amniotic cell cultures had CD90 and CD73 markers on the cell surface, while a negligible number of cells expressed CD105 and HLA-ABC markers (also characteristic of MSC) [68].

In the early 2000's, the immunogenic characterization of hAM was still controversial, and the use of immunocompetent animal models raised new questions. Xenotransplantation of cryopreserved hAM to the limbal area, intracorneal space, and under the kidney capsule in rats showed good tolerance of this immune-privileged xenograft in all the sites [12]. At the same time, the histological analysis revealed that HLA-A, -B, -C and -G proteins were frequently expressed in epithelium, mesenchymal cells, and fibroblasts in the cryopreserved hAM [12]. Class I Ag remained strongly expressed even after 6 months' cryopreservation. Hori et al. focused on the immunosuppressive and immunogenic potential of fresh hAECs in a murine xenotransplantation model [69]. They concluded that fresh amniotic epithelium expresses HLA class I Ags and sensitizes rat recipients when placed in the eye, although long-term memory of allospecific delayed-type hypersensitivity was not acquired. They stated that amniotic epithelium was clearly vulnerable to acute immune rejection in specifically sensitized recipients and/or recipients of repeated amniotic epithelium transplants. They emphasized that the immunogenicity of amniotic epithelium should not be ignored, and that hAM from different placental donors should be used when repeat transplantation is required in patients.

Similarly, other authors reported that fresh amniotic epithelium was immunogenic when placed on the ocular surface in a murine allotransplantation model, although no memory of allospecific delayed-type hypersensitivity was acquired [70]. They postulated that allogeneic amniotic epithelium is clearly vulnerable to immune rejection in specifically sensitized recipients. They suggested that amniotic epithelium is not a completely immune-privileged tissue, displaying immunogenicity after transplantation into a normal mouse eye and acting as a rejection target in the eyes of presensitized recipients. On the contrary, in 2011, successful xenotransplantation of cryopreserved hAM on rat abdominal wall muscles (i.e., unprivileged tissue) was reported [71]. There was no graft rejection, and the immunological response to hAM was reduced by using a multilayered patch and cyclosporine as an immunosuppressor.

## 4 Conflicting historical data on hAM inflammation/immune reaction

In 2000, Gabler et al. initially reported that a patient who received a corneal hAM cryopreserved transplant from the same donor on three separate occasions developed a hypopyon (polynuclear accumulation in anterior chamber) after the second and third applications [72]. It is in this context that the two aforementioned studies in animal models were conducted to explore the underlying mechanisms, leading the authors to suggest that mild or transient sensitization could occur, depending on the implantation site [69,70]. Similarly, four additional cases of hypopyon were described after cryopreserved hAM was used as a first graft or after a second graft in the cornea [73,74] as well as after fresh hAM transplantation in the cornea [75]. In all these studies, the authors suggested that the patient may have developed an immunological response to that donor's tissue. Although the occurrence of hypopyon after hAM grafting remains rare, we hypothesize that the patient's immune response to the causative infection may be enhanced by the immunomodulation effect of the hAM, and we encourage future investigations.

More recently, a systematic review and meta‐analysis studied hAM graft rejection based on clinical signs [76]. The authors mentioned three human studies in which no signs of rejection were identified [[77], [78], [79]]. But it is important to note that graft rejection was evaluated with (hyper)dried hAM with no remnant viable cells [77,79] or in an original way in autologous use [78]. Additionally, the authors reported data on graft rejection from seven animal studies [[80], [81], [82], [83], [84], [85], [86]]. No meta-analysis was conducted for this outcome since only one study quantified the rejection response, showing subtle transient transplant glomerulitis between 3 and 6 weeks, but no other signs of transplant rejection [80]. According to our analysis, no signs of graft rejection were reported in all these animal studies when hAM was grafted into immunologic unprivileged site in fresh [81,83,84], cryopreserved [85], dried [80], decellularized [82], or glutaraldehyde-preserved [86] formats.

## 5 Key factors affecting hAM immunogenicity

In routine use, hAM that is cryopreserved, or hAM that is lyophilized/dehydrated ± decellularization ± gamma sterilization, has a good clinical safety profile, as serious adverse reactions and immunological or inflammatory responses are rarely observed despite its extensive clinical use [6,[13], [14], [15], [16], [17], [18]]. While it has been used in 22,776 ophthalmic grafts across Europe in 2022 [13], neither the persistence of viable cells just before the grafting nor potential hAM immunogenicity after transplantation have been thoroughly investigated [19,[87], [88], [89]]. These knowledge gaps are particularly relevant given that factors such as graft format and clinical indications (application site) may influence the immunogenic behavior of hAM [19,90].

a) hAM graft format

Based on our analysis of the literature, the effect of cell (or residual cell) viability in fresh or processed hAM formats on hAM immunogenicity has not been examined.

Fresh hAM – which is assumed to contain viable cells – was not involved in any acute immune reactions, either in allogenic situations or in xenografts, leading some authors to conclude that residual living amniotic cells in whole tissue are involved in immunomodulation and immunoregulation [7,8,[91], [92], [93], [94]].

Since cryopreservation was introduced for hAM storage, the presence of residual viable cells remains controversial [3,12,[95], [96], [97]] and depends on the method used [[98], [99], [100]]. Studies, including our own [96], have shown a significant decrease in cell viability compared to fresh hAM. Thus, we questioned the relevance of assessing residual cell viability for clinical applications [101]. While cryopreservation preserves extra cellular matrix (ECM) integrity and bioactive factors, the limited post-thaw viability of cells reduces their contribution to immunomodulation [98,102]. However, this is partially offset by the preserved soluble factors and matrix-associated molecules that retain substantial immunomodulatory activity [15,66,103,104].

Additional processes have been developed to improve hAM storage and safety. Lyophilization, often combined with gamma irradiation, can induce significant structural and biochemical alterations in hAM, including changes in ECM organization and reductions in certain growth factors [1,19,90,105,106]. Despite these changes, key bioactive components are partially preserved, allowing the membrane to retain at least some of its immunomodulatory activity. Gentler sterilization methods are recommended to preserve its immunomodulatory and regenerative activity [19,107,108]. Dehydration affects mechanical properties and total growth factor levels but does not alter immunogenicity [19,90,105]. Overall, both lyophilization and dehydration have minimal impact on hAM immunogenicity due to the preservation of key structural and bioactive components. Decellularization processes, which were developed to prevent graft rejection [109], have been applied to hAM with claims that they ensure its safety and prevent graft rejection [19,110,111], although some authors have questioned its complete immune tolerance. [112,113]. While this process preserves the ECM scaffold, it results in significant loss of growth factors and immunomodulatory proteins [21,90]. Harsh decellularization processes can damage the ECM structure, expose cryptic antigenic sites (particularly in collagen), and release damage-associated molecular patterns (DAMPs). These can trigger innate and adaptive immune responses and limit immune tolerance [88,89]. Mild decellularization protocols that preserve ECM integrity while removing cellular Ags maximize immune tolerance. A variety of factors have been demonstrated to influence host immune responses, including residual DAMPs, cytotoxicity, scaffold origin, and implantation site [30,31]. Furthermore, studies on electrospun scaffolds incorporating decellularized hAM-derived ECM have demonstrated both low immunogenicity and significant immunomodulatory effects, including regulation of macrophage phenotype, suppression of inflammatory responses, and promotion of tissue regeneration [114,115].

The hAM graft format is also subject to significant intra- and inter-donor variability, which affects its biological properties, including growth factor content and regenerative potential [19,116,117]. However, hAM immunogenicity is rarely assessed with respect to donor variability, leading to batch-to-batch heterogeneity that can impact therapeutic efficacy.

b) Significant role of hAECs

Based on our analysis of the literature, the role of hAECs on hAM immunogenicity has not been defined.

Akle's seminal publication focused on hAECs/amniotic epithelium but did not evaluate cell viability [10]. Later, several studies showed that HLA Ags are frequently found in this layer [60,65,69,70]. This may partly explain Akle's focus on these specific cells, as the amniotic epithelium appeared immunologically active. However, subsequent work has refined this view, showing that despite detectable HLA expression, hAECs display a remarkably low immunogenicity risk profile and a strong capacity for immune regulation [52,118].

Beyond their immune phenotype, hAECs have attracted attention because of their embryonic lineage and the expression of pluripotency-associated transcription factors. such as OCT-4, SOX-2, NANOG, and SSEA-4, which confer broad developmental plasticity [119,120]. In culture, hAECs can undergo epithelial-to-mesenchymal transition, acquiring mesenchymal-like features and differentiating toward multiple lineages, including osteogenic, adipogenic, hepatic, and neural fates [[119], [120], [121]]. In particular, when the intact hAM was cultured in osteogenic conditions, hAECs had a mesenchymal phenotype and produced hydroxyapatite, confirming that the osteogenic potential of the membrane resides mainly in its epithelial layer [92]. Furthermore, hAECs retain viability, stemness, and metabolic activity after cryopreservation, while preserving their paracrine and differentiation capacities—an essential feature for biobanking and clinical-scale applications [122,123].

In clinical practice, the presence or absence of the hAEC layer profoundly influences the membrane's function, making the choice between intact and de-epithelialized (or denuded) hAM dependent on the intended regenerative or immunomodulatory outcome. Denuded hAM, from which the epithelial layer has been removed, is widely used for ocular surface reconstruction, particularly in limbal stem cell transplantation, because it promotes efficient epithelial cell adhesion, stratification, and differentiation [104,124]. In contrast, intact hAM (retaining its native epithelial layer) remains a valuable surgical material when applied to bare sclera to inhibit conjunctival overgrowth [104,124].

Altogether, these findings highlight the dual identity of hAECs as both a biologically versatile and clinically tractable cell population within the hAM.

c) Implantation sites or clinical indications

Based on our analysis of the literature, hAM immunogenicity can vary depending on whether it is applied to immune-privileged or non-privileged sites.

In the historical dermatological application, where the graft is applied to immunologically active tissues such as the skin, hAM demonstrates excellent tolerance regardless of its preparation (fresh, preserved, epithelialized or denuded) [7,17,[125], [126], [127], [128], [129]].

In ophthalmology, hAM is applied to both immune-privileged sites (e.g., cornea, anterior chamber) [3,130] and non-privileged areas (e.g., conjunctiva, retina) [131,132]. Despite this diversity in graft location and vascularization, clinical outcomes consistently show minimal (or even absent) inflammatory reactions and no significant rejection. Only a few clinical cases of post-hAM graft inflammation have been reported in the literature: a hypopyon following one or repeated hAM applications on the ocular surface in the same patient [[72], [73], [74], [75]]. Interestingly, xenogeneic hAM grafts in the cornea exhibited no immune response and maintained transparency long-term, reinforcing the notion of an intrinsic immunomodulatory property [12].

Furthermore, hAM has regenerative and anti-fibrotic effects in preclinical models, reducing liver fibrosis in rats [133,134], and promoting cardiac repair with decreased fibrosis and enhanced cardiomyocyte survival in ischemic rat hearts [135]. It has been also applied in a variety of clinical contexts, including wound healing, chronic ulcers, burns, prevention of adhesions and scarring, ophthalmology, gynecology, plastic and reconstructive surgery, gastrointestinal and trauma surgery, neurosurgery, oral and dental applications [9,90].

Therefore, there is consistent clinical and experimental evidence that hAM integrates well without rejection, indicating that its immunotolerance relies not solely on the graft site's immune status but also on intrinsic properties that promote tolerance across diverse tissues.

## 6 Importance of re-evaluating hAM immunogenicity

To summarize, the production of anti-HLA Abs after hAM transplantation has not been investigated since the Akle study in the 1980s [10], despite the advent of newer and much more sensitive detection techniques. In addition, that study explored the immune response after subcutaneous hAM transplantation, which is not a clinical indication. Nevertheless, that study has been cited nearly 724 times and still constitutes, for the scientific community, proof of the absence of HLA-mediated immunogenicity of the hAM. None of the previous studies quantified the immune response, and the experimental conditions varied widely, including hAM format and residual cellular or DNA content, graft site immune privilege, species differences, and assay sensitivity.

hAM transplantation is a rare example of a massive allogenic input of HLA-G. Yet, anti HLA-G immunization following this procedure has never been investigated. Contrary to organ and hematopoietic stem cell transplantation, HLA Abs screening before and after hAM transplantation is not a standard procedure. It is not performed in France, and its research is country dependent. Additionally, no European or international guidelines exist on this subject.

Importantly, the non-immunogenicity of hAM has been primarily demonstrated clinically, through the consistent absence of graft rejection, which ultimately validates its safety. For these reasons, we feel that despite the excellent clinical tolerance after hAM transplantation, its immunogenicity should be reassessed in the context of the current clinical use of hAM, especially when hAM is grafted into deep and vascularized tissues [136,137], as these are more conducive to the appearance of a *de novo* immune response, or in patients with prior immunization which could negatively impact the graft's efficacy. The goal is to provide biological confirmation of the humoral immunotolerance of hAM grafts, supporting the extension of its recognized safety to other potential indications, particularly in vascularized and deeper tissues.

This led us to revisit the potential development of anti-HLA class I and II Abs after hAM transplantation using the modern gold standard method (Luminex®). Since there is no commercial kit available to detect anti-HLA-G Abs, we adapted the MAIPA ApDIA® assay, which we have previously validated and published for anti-HLA-G Ab detection.

This perspective article builds on preliminary data from a clinical study using a glycerol-cryopreserved hAM routinely applied in ophthalmology – the only indication currently authorized in France [138,139] – for which cell viability, although not routinely assessed in the French tissue bank, has been characterized in our previous studies [96].

## 7 Methods for HLA class I and class II detection in patient sera

a) Luminex® multiplex assay

The conventional and historical assay for detecting anti-HLA Abs is the micro-lymphocytotoxicity test, which is done by directly measuring complement-dependent cytotoxicity (CDC) on viable peripheral blood lymphocytes. In the early 2000s, techniques using Luminex® equipment became the gold standard for monitoring patients awaiting transplantation and during the post-transplantation period. The exploration of anti-HLA Abs is carried out in two steps—screening and identification—with the two available kits being very similar conceptually.

Briefly, the sample is incubated with a mixture of fluorescent polystyrene microbeads, each coated with different HLA class I or class II Ags (Ag extract pools for screening or recombinant Ags for identification). After the beads are washed through a filter plate, Ab binding is revealed by fluorescent secondary Abs. The bead suspension is then analyzed in a flow cytometer. Two lasers identify each bead through its intrinsic fluorescence and quantify the signal level linked to the quantity of Abs fixed on it [140].

This technique has major advantages. First, various classical HLA class I and class II molecules can be analyzed at the same time using a very small volume of serum or plasma. Second, the mean fluorescence intensity (MFI) measured for each bead is a quantitative signal whose value is presumed to be proportional to Ab level in patient sera, obtained for single Ags, well identified at the allelic level. Therefore, it is commonly used to determine and follow a patient's level of immunity. Lastly, the sensitivity is very high: the MFI threshold predictive for a positive CDC reaction varies according to the locus, but is at least 9000 for locus B, whereas the commonly accepted positivity threshold for this technique is around 1000–2000, or even less [141].

This technique also has its drawbacks. The first is the prozone phenomenon: complement fixation following Ab fixation on the Ag coated on the beads’ surface prevents fixation proportional to the concentration of Abs present in the sample due to steric hindrance [142]. Consequently, it can artificially diminish the MFI and underestimate the true immunity level. Fortunately, this can be easily remedied by heat treatment or by adding EDTA in the serum before the assay. Second, Luminex® can detect HLA class I Abs even in non-alloimmunized healthy males [143], consequently considered as “natural” Abs. HLA cryptic epitopes can be exposed following Ag denaturation due to β2m loss during the assay process [144]. Cross-reactivity with epitopes of other molecules such as bacterial or animal proteins or non-classical HLA molecules may explain why these natural HLA Abs are present even without a previous immunization event [145]. Natural Abs against HLA class II Ags have also been reported [146].

b) Modified MAIPA (Monoclonal Antibody-specific Immobilization of Platelet Antigens) assay

Multiplex Luminex® was used in a recent study to screen sera for Abs directed against non-classical HLA (HLA-G, -E and –F) and β2m [145]. HLA-G molecule was purified and coated on Luminex beads. Interestingly, anti-HLA-G IgG Abs were detected in 6 of 17 healthy subjects and were more frequent than those directed against HLA-E and HLA-F. In addition, anti-HLA-G levels were stable in 5 individuals over 6 months. However, this study has a technical limitation: during the production of the beads and the coating process, HLA-G molecules can be damaged and cryptic sites exposed due to loss of β2m. Therefore, nonspecific Ab binding may occur, resulting in false positive anti-HLA-G Abs detection.

**Fig. 2:**
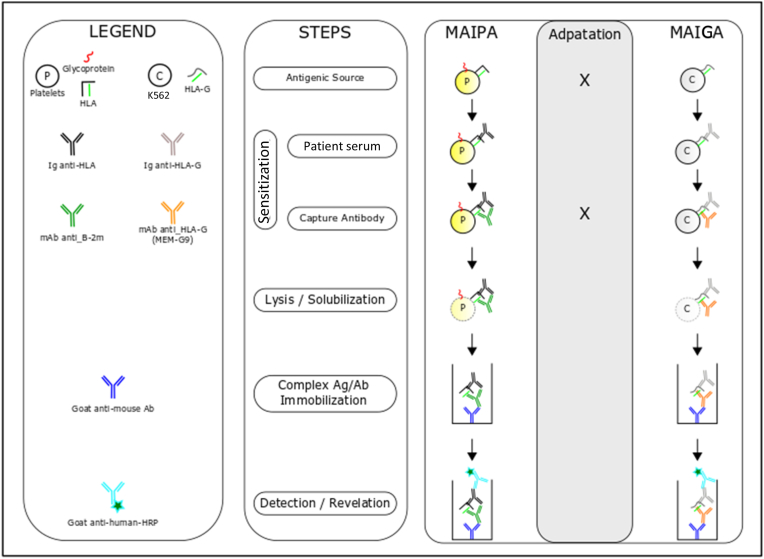
Summary of the differences between the original MAIPA ApDIA® assay and our modified MAIPA assay adapted from Pedini et al. [148]. Transfected K562 cells overexpressing HLA-G are used for detection and untransfected K562 cells (wild type) for negative controls. Cells are incubated with a patient's serum. Then MEG/9 (mouse anti HLA-G) is used for capture. After cell lysis, the complex is immobilized using coated goat anti-mouse antibody. Finally, anti HLA-G antibody is revealed using goat anti-human HRP.

To reduce this risk of false positives, we used a different detection approach. We modified the MAIPA ApDIA® kit to detect Abs directed against HLA-G associated with β2m [147]. This kit is based on the ELISA principle and allows the detection and identification of anti-platelet glycoprotein auto- and allo-Abs and the detection of anti-classical class I HLA Abs in serum using a platelet panel and platelet-Ag immobilized by a monoclonal Ab [147]. Its sensitivity is similar to Luminex® for the detection of antiplatelet Abs and for classical HLA class I Ags [147]. All the MAIPA reagents were unchanged from our modified protocol except the platelet panel and capture Ab. These were replaced by K562 transfected cells expressing different HLA-G variants as a control and by MEM-G/9 monoclonal Ab as a capture Ab (Fig. 2). Untransfected K562 cells were used as negative controls and tested for each serum sample. This method has been validated for HLA-G Ab detection and published in a study involving a lung transplant cohort [148].

## 8 Conclusion

The current literature on hAM immunogenicity is sporadic and sometimes contradictory. This may be due to differences in: (i) hAM formats (with residual viable cells or DNA debris), (ii) hAM grafting sites (immune-privileged or not), (iii) species and/or (iv) assay sensitivity.

The production of anti-HLA Abs following hAM transplantation has never been determined in clinical applications using modern techniques. We propose to look for the development of anti-HLA class I and II Abs post-hAM transplantation using the modern gold standard method (Luminex). Additionally, we want to assess anti-HLA immunization more comprehensively by using an adaptation of the MAIPA ApDIA® assay, which we have previously validated for anti-HLA-G Ab detection.

We are not questioning the immunogenicity of hAM. Instead, we want to document it more thoroughly and provide biological confirmation of its humoral immunotolerance under the conditions in which hAM is currently being used. We believe it is crucial to better understand the hAM's potential to trigger the production of specific antibodies in the context of subsequent transplants or pregnancies. Our work should reinforce the current clinical use of hAM and expand its applications, especially in vascularized, deeper tissues.

## CRediT authorship contribution statement

**Jean-Baptiste Baudey:** Writing – review & editing, Methodology, Investigation, Formal analysis, Data curation, Conceptualization. **Lauriana Solecki:** Writing – review & editing, Writing – original draft, Visualization, Validation, Methodology, Investigation, Formal analysis, Data curation, Conceptualization. **Christophe Picard:** Writing – original draft, Formal analysis, Data curation, Conceptualization. **Pascal Pedini:** Writing – original draft, Formal analysis, Data curation, Conceptualization. **Bastien Mathéaud:** Methodology, Investigation, Funding acquisition, Formal analysis, Data curation, Conceptualization. **Alain Coaquette:** Methodology, Investigation. **Isabelle Jollet:** Methodology, Investigation, Funding acquisition, Formal analysis, Data curation, Conceptualization. **Pauline Jamain:** Methodology, Investigation, Funding acquisition, Formal analysis, Data curation, Conceptualization. **Lucas Hubert:** Methodology, Investigation, Formal analysis, Data curation, Conceptualization. **Adeline Desanlis:** Visualization, Methodology, Investigation. **Xavier Lafarge:** Writing – original draft, Visualization, Validation, Methodology, Investigation, Formal analysis, Data curation, Conceptualization. **Florelle Gindraux:** Writing – review & editing, Writing – original draft, Visualization, Validation, Supervision, Project administration, Methodology, Investigation, Formal analysis, Data curation, Conceptualization.

## Ethical approval

Not applicable.

## Informed patient

Not applicable.

## Funding

Call for proposals 2023, French Agency of Biomedicine.

## Declaration of competing interest

The authors declare that they have no known competing financial interests or personal relationships that could have appeared to influence the work reported in this paper.

## Data Availability

No data was used for the research described in the article.
